# Longitudinal associations between cognitive ability and socioeconomic status are partially genetic in nature

**DOI:** 10.1038/s41598-026-37786-3

**Published:** 2026-02-02

**Authors:** Petri J. Kajonius

**Affiliations:** Department of Psychology, Lund University, Lund, Sweden

**Keywords:** Cognitive ability, IQ, Intelligence, Socioeconomic status, SES, Twins, Genetics, Psychology, Psychology

## Abstract

Individual’s future socioeconomic status (SES) has been reported to be robustly predicted by cognitive ability (IQ). However, research on the genetic and environmental underpinnings of this association in emerging adults remains limited. Utilizing the German TwinLife panel data, the present study examined how IQ at early adulthood at age 23 is associated with SES at age 27 (N_MZ_ = 228 and N_DZ−SAME SEX_ = 212), through 2 measures on educational attainment and 2 on occupational status. Cholesky decomposition models reported the heritability of IQ at approximately 75%, and heritability on all SES outcomes. Genetic factors further explained most of the IQ–SES association (69–98%), and genetic correlations between IQ and SES exceeded environmental correlations. These findings seem to underscore the importance of researchers and policymakers to also considering genetic factors when examining the life outcomes of young adults.

**Supplementary Information:**

The online version contains supplementary material available at 10.1038/s41598-026-37786-3.

One of the more relevant topics in social science is arguably the psychological mechanisms behind socioeconomic status (SES), in the present study defined and measured through educational attainment and occupational prestige. In emerging adulthood, getting an education and getting a job are arguably the primary means to start making living income. A near consensus in the research literature is that general cognitive ability, defined and operationalized through the sum of various cognitive tests, measured as the Intelligence Quotient (IQ), is the strongest predictor for SES; Longitudinal phenotypic studies (k = 65) report effect sizes of around *r* = .50 in meta-analysis^1^. What is still less clear is to what extent this relationship is driven genetically or by the individual’s unique personal experiences, for instance through social networking in higher studies or job market advancements. The present study aimed in a basic way to analyze how much one’s genes account for the longitudinal impact of IQ on SES in emerging adulthood (a brief period here defined as ages 23 to 27), based on representative panel data from TwinLife, Germany. The present study is among the first to report on the size of longitudinal genetic and environmental correlations (n.b., mutually explained variance between IQ and SES), based on young adult population data.

## Cognitive ability and socioeconomic status

Cognitive ability through IQ is reported to phenotypically and longitudinally predict future socioeconomic status (SES), even reported to compensate for lower family SES^2^, based on a representative US sample. Individual level cognitive ability in emerging adulthood is reported to be one of the most important mediators of family SES for one’s future educational and occupational status^3^. Genetically informed research using DNA from unrelated representative UK children (*N* = 3,000) reports near perfect genetic overlap between family SES and child cognitive ability (r_G_ = 1.0), measured at ages 7 and 12^4^. However, studies on very young children often come with large uncertainties and may be confounded by unmodeled environmentally mediated effects. Also, IQ heritability, i.e. the proportion of individual differences in IQ which can be attributed to genes, may be as low as 20% in early childhood, rising to 80% in late adulthood^5^. This could among other things suggest continual activation of individual genes and increasing person-environment fit throughout adulthood^6^. Learning environments and practice influences on IQ are also ever relevant, such as intellectual climate at home^7^ or additional years of higher education, according to meta-analysis^8^.

Similarly, SES is also partly genetic in origin. A recent Norwegian twin panel study reported on five measures of SES, quantifying the genetic component to 34%–47% and the shared family environment to 16%^9^ The remainder of environmental variance usually consist of largely individual experiences and measurement errors. Genetic transmissions in SES have also shown to be causal in five longitudinal population DNA-based studies, reporting that higher polygenetic scores differing from parents and siblings also lead to higher SES^10^. This seems to concur with ambitious analyses of over 400 years of family tree lineages in the UK, where social outcomes report genetic correlations across generations all the way to 4th cousins^11^. More remarkably, the size of correlations based on genetic distances are reported to be robust across the varying cultural eras^11^.

In summary, individual IQ and SES seem dependably correlated, and both heritable. The objective of the present study, based on emerging adult population twin panel data, was to complement the body of literature on the subject by quantifying simplistically how much of the association of IQ on SES is transmitted via genes, and to what extent these are the same for IQ and SES (i.e., overlapping genetic variance). This would further psychological and social research, including policy, law, and politics, as well as inspire researchers to control for individual psychological factors.

## Method

### Procedure and sample

The sample in this study was based on the TwinLife project, Leibniz Institute for Social Sciences (GESIS.org). TwinLife is a longitudinal twin family study which incorporates over 4,000 German families, with the aim of studying social inequalities from genetic and psychosocial perspectives over the life course^12^. Monozygotic twins and same-sex dizygotic twins were interviewed face-to-face at the start in 2014 and continuously every other year, together with supplementary phone-based surveys. Zygosity was collected through a physical similarity questionnaire, and comparisons to DNA tests have a reported accuracy rate of 92–96% (See all details in^13^.

The data was representative of all regions of Germany, with an overall response rate of 37%. 57% of respondents were women, German citizenship 85%, and university educated households was 44%, slightly higher than German representative data; No systematic dropout bias in the TwinLife data has been reported on personality, relational characteristics, age, cohort, and sex^13^. Only people over 20 and below 30 years of age (M_age_ = 23.1, SD = 1.8 at first wave and M_age_ = 27.2, SD = 1.9 at the second wave) were analyzed (N_Total_ = 880, after pairwise deletion, *N* = 583—812, See Supplemental Materials Table S1). The twin pair distribution was: N_MZ_ = 228 and N_DZ−SAME SEX_ = 212.

### Instruments

#### Cognitive ability (IQ)

Cognitive ability was measured at Wave 1 age 23 using Cattell’s Fluid Cognitive ability Test (CFT-20-R^14^. The CFT-20-R is comprised of four subtests (figural reasoning, figural classification, matrices and reasoning) with 56 items, with increasing difficulty. For this study, correct answers were only counted if given within the predetermined time limit of each question. The test was administered in a computer-based format. The Chronbach’s alpha coefficient was α = 0.80^15^.

#### Education

Educational level was measured by the ISCED (International Standard Classification of Education) which is a system comprised of levels classifying education by complexity (OECD, 1999^16^. A second TwinLife measure for educational level was Casmin (Comparative Analysis of Social Mobility in Industrial Nations). Both scales were analyzed on a 0–10 scale, where a zero is typical of kindergarten-level education while the highest equals a doctorate degree.

#### Occupation

Occupation was also measured with two diverse variables due to the complexity of occupation rankings. Occupational prestige was measured using the SIOPS (Standard Index of Occupational Prestige Scale^17^ ranking from 0 to 100 based on the participants’ current position, which in turn was based on classifications from the ISCO-08 (International Standard Classification of Occupations^18^. A higher number indicates higher occupational prestige. A second measure, Occupation socioeconomic status was measured using the ESeC (European Socio-Economic Classification) which categorize occupations based on their position on the labor market. This was analyzed with ordinal scale ranging from 1 to 9 where a higher number translates to occupational positions with higher autonomy and socioeconomic status. See Supplemental Materials Table S1 for skewness and kurtosis.

## Ethics

The TwinLife project was ethically approved by the German Psychological Association (RR 11.2009 and RR 09.2013). Participants were informed of the study aim, data protection regulations and their right to withdraw at any time. All collection and analysis of the data was performed in accordance with regulations, guidelines, and ethical approval. Informed consent was obtained from all the participants for study participation.

### Statistical analysis

First, longitudinal zero-order within-person correlations between IQ at age 23 and the SES outcomes at age 27 were analyzed to establish concord with the prevailing literature on socioeconomic outcomes. All study variables were then residualized for sex and age in preparation for analyses in a structural equation model (SEM) framework. Missing data was handled through pairwise deletion, based on non-significant MCAR testing (missing at random). All subsequent analyses were conducted in AMOS v.23.

Second, univariate classic twin design (CTD) models were conducted between twin pairs, one for each study variable, for the purpose of determining best fitting baseline models for genetic and environmental analyses. The CTD decomposes variables’ variance into three latent factors for each twin, estimating: (A) additive genetics, constrained between monozygotic twins (MZ) as 1 (sharing identical genes) and between dizygotic twins (DZ) as 0.5 (sharing on average half of genes), (C) common family variance, constrained to 1 for all twin pairs (cf. Equal Environment Assumption), and (E) the environment, unique for every twin. Model fit estimates in CTD determine whether it is most effective to decompose the variable variance into ACE or only AE models. ADE models, quantifying dominance (D) gene expressions instead of common (C) family variance, even though feasible and interesting, were not included in the basic present study objectives. Furthermore, most variables showed less than double the correlations for MZ compared to DZ. Several diverse model fits were chosen for comparison, Chi^2^ (significance testing), AIC (lower the better), and RMSEA (as close to 0 the better). CTD estimates heritability by squaring the (A) standardized estimate.

Third, bivariate longitudinal Cholesky models were computed, with SES at age 27 as the dependent variable, regressed on IQ at age 23 as the predictor, one model for each of the 4 SES outcome measures. See Fig. 2 for visualization of the Cholesky model. These were conducted in multigroup SEM, one group for each twin, constrained in-between twins, in the same way as in CTD. The Cholesky models enabled not only estimation of heritability (A23 in Fig. 2), but how much of the variance between IQ and SES measures were genetic and environmental, as well as how much of variance in SES measures was *not* explained by IQ (A27 and E27 in Fig. 2). Furthermore, Cholesky-based genetic correlations, which is the amount of overlapping genetic (co)variance between IQ and SES in relation to total genetic variance, could be calculated with the formula r_G_ = A_IQ23−SES27_/√(A23*A27)^19^ – See Fig. 2. See^6^ for a detailed overview of twin analyses.

## Results

Descriptive statistics of the study variables, including means and distributions, are found in Table S1 in Supplemental materials. The zero-order correlation matrix between study variables based on within-person waves of measurements is presented in Table 1. IQ at age 23 correlated with both SES measures of education and both SES measures of occupation at age 27 in the higher range (*r* >.30; Using guidelines from^20^.

**Table 1:** Correlational matrix study Variables.

1.IQ23_Cognitive ability	1	2	3	4
	—			
2.SES27_Edu. Level	0.34	—		
3.SES27_Edu. Casmin	0.43	0.88	—	
4.SES27_Occ. Prestige	0.27	0.43	0.47	—
5.SES27_Occ. SES	0.31	0.48	0.48	0.73

Note. df = 513—730. All estimates were significant at *p* <.001. Confidence intervals at 95% were between +−0.06 to +- 0.08 around the estimates. Example *r* =.48 reported 95% CI [0.41, 0.54]. 23_ and 27_ indicate at what age the individual was measured. See Supplemental Materials Table S2 for all estimates.

Univariate Classic Twin Design model (CTD) was applied for each of the study variables to determine best fitting baseline models for the genetic and environmental Cholesky analyses. The first column in Table 2 presents intra-class correlations overall being higher for monozygotic than dizygotic twins, implying genetic explanation (A), but for most also less than double the size, implying no non-additive dominant genetic variance. Therefore, only the basic additive ACE and AE models, with and without common (C) family environment, were compared. In overview, Falconer’s formula (A = 2 x (r_MZ_-r_DZ_)) can be used to approximate heritability^9^. This resulted in, for example, a high 88% heritability for IQ.

In the next column in Table 2, Chi-squared-testing confirmed overall insignificance (with one exception for Occupation Prestige), and model fit statistics AIC and RMSEA established the more parsimonious AE models overall fitting the data better than ACE (with the one exception for Occupational SES). The heritability for each of the study variables, based on the CTD models, were 75% for IQ, 49% and 66% for education, as well as 32% and 71% for occupation. Study variables reported on average 44% genetic (A) explanation. The AE models suggested common (C) family environment variance on average of 25%, however, model fits indicated that this variance overall was more appropriately modelled as genetic variance. Note that the p-value should *not* be significant, but in light of a larger sample the Chi-square test is often too sensitive^21^. The remaining variance (E) usually consists of unique life experiences, randomness in events, and measurement error (unreliability).

**Table 2:** Univariate classic twin models for study Variables.

Variable	(*r*_MZ_/*r*_DZ_)	Model	Chi2	df	*p*	AIC	RMSEA	A %	C %	E %
IQ23_Cognitive ability	(0.76/0.32)	ACE	4,973	3	0.17	18,973	4,973	75	0	25
		AE	4,973	4	0.29	16,973	4,973	75	-	25
SES27_Edu. Level	(0.47/0.35)	ACE	2,275	3	0.52	16,275	2,275	29	19	52
		AE	3,643	4	0.46	15,643	3,643	49	-	51
SES27_Edu. Casmin	(0.62/0.47)	ACE	5,365	3	0.15	19,365	5,365	42	22	36
		AE	3,996	4	0.41	15,996	3,996	66	-	34
SES27_Occ. Prestige	(0.59/0.46)	ACE	7,623	3	0.05	21,623	7,623	23	35	42
		AE	7,886	4	0.01	19,886	7,886	32	-	68
SES27_Occ. SES	(0.59/0.46)	ACE	1,681	3	0.64	15,681	1,681	31	39	30
		AE	6,212	4	0.18	18,212	6,212	71	-	29

Note. N_pairs_ = 282—380. Confidence intervals at 95% for r_MZ_ and r_DZ_ were between +−0.07 to +- 0.15 around the estimates. IQ23_ = Cognitive ability measured at age 23. SES27_ = Socioeconomic status measured at age 27. r_MZ_ = intra-class correlation between monozygotic twins. r_DZ_ = intra-class correlations between dizygotic twins. Chi^2^, AIC and RMSEA = Model fits. A = Additive Genetics, C = Common Environment (Home/School), E = Unique Environment.

**Fig. 1:**
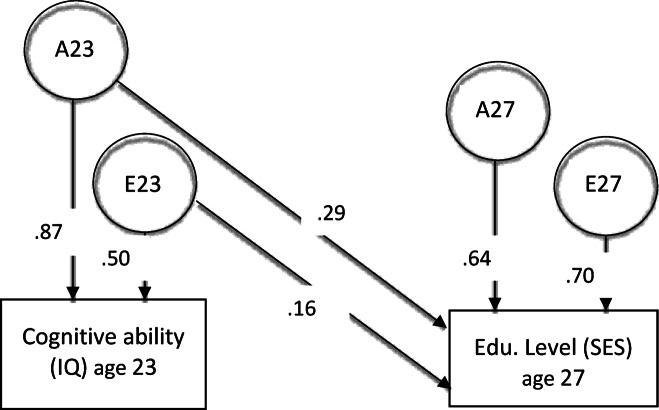
Bivariate Longitudinal Cholesky Model of Cognitive ability (IQ) and Socioeconomic Status (SES). Note. All estimates were standardized. N_twinpairs_ = 370. Confidence intervals at 95% were +−0.09 around the estimates. Cognitive ability predicted socioeconomic status (here illustrated by Educational Level), decomposed into genetics and unique environment variance. See Table 3 for all model results. All estimates were squared for % explained variance: For example, 41% (0.64 squared) of Education heritability at age 27 was *not* explained by IQ genes. A = Additive Genetics, E = Unique Environment. (C) Common home environment was not modeled due to worse model fits (Table 2). Only one twin was visualized for simplicity in the MZ/DZ-constrained multigroup Cholesky model.

Bivariate longitudinal Cholesky models were fitted between IQ at age 23 and each one of the 4 SES variables at age 27, as illustrated in Fig. 1. The results, including model fits and estimates, are presented in Table 3. The last four columns contain the study objectives. The share of genetic IQ variance explained 69% and 81% of the total phenotypical relationship with educational SES, and 98% with occupational SES – overall multiple times higher than the environmental explanations. Furthermore, the genetic correlations, that is the amount of genetic covariance between IQ and SES (r_G_ = A_IQ23−SES27_/√(A23*A27)), were overall high and more than double the environmental correlations, equaling multiple times the explained variance (comparing the square of the correlational coefficients).

**Table 3:** Bivariate longitudinal cholesky models of cognitive ability (IQ) and socioeconomic status (SES).

Models	Chi^2^	*p*	AIC	RMSEA	A23 -> SES27	E23 -> SES27	A27	E27	% gene	% env.	*r*_G_	*r*_E_
					Gene IQ	Env IQ	GeneSES	Env SES				
IQ23 -> SES27 Edu. Level	12,871	0.46	41,178	0.00	0.29	0.16	0.64	0.70	0.69	0.31	0.43	0.23
IQ23 -> SES27 Edu. Casmin	17,480	0.18	45,492	0.028	0.41	0.14	0.69	0.57	0.81	0.19	0.59	0.22
IQ23 -> SES27 Occ. Prestige	24,481	0.03	52,481	0.045	0.30	0.01	0.71	0.64	0.98	0.02	0.42	0.01
IQ23 -> SES27 Occ. SES	33,343	0.00	61,344	0.06	0.31	0.01	0.78	0.54	0.98	0.02	0.42	0.02

Note. N_twinpairs_ = 282—380. See Figure 1 for illustration of the Cholesky model and variable labels. IQ23 = Cognitive ability measured at age 23. SES27 = Socioeconomic status measured at age 27 (4 outcome models). A23 and E23 = Genetic and Environmental estimates of IQ at age 23, which for all models were 0.87 and 0.50 (Fig. 1). A27 and E27 = Gen and Env. residual estimates at age 27 not explained by Cognitive ability IQ. Chi^2^, AIC and RMSEA = Model fits. df = 12 for all models. All estimates were standardized. % gene = percentage of total phenotypical shared variance explained by genetics, % env = percentage of total shared phenotypical variance explained by environment. r_G_ = genetic correlation (i.e., overlap genes). r_E_ = environmental correlation (i.e., overlap experiences). Grey columns were the study objectives.

## Discussion

Based on a representative panel of emerging adults, individual cognitive ability (IQ) reported to be a reliable predictor for socioeconomic status (SES), in terms of educational attainment and occupational prestige. Both IQ and SES were heritable, with commonly more than half of the variance between individuals explained by their genes, and not as much by common or unique life experiences. Similarly, the longitudinal *associations* of IQ on future SES were mostly explained by genetics, and not as much by environmental influences. Moreover, not frequently reported based on representative panels before, IQ and SES reported overlap in the form of genetic correlations (shared explained genetic variance), explaining multiple times more variance than the environmental correlations.

These genetic influences could operate in several different ways. First, the same genes can simultaneously affect brain development (and thus IQ) and traits or behaviors that promote socioeconomic success, creating a correlation without IQ itself being the cause of SES (direct or biological pleiotropy). Second, genes could enhance IQ, and that higher IQ could then causally open doors to higher education, better jobs, and upward social mobility (mediated pleiotropy). Both mechanisms likely have contributed: part of the IQ–SES link is direct (shared genes), and part is indirect (genes → higher IQ → higher SES). In addition, it is also appropriate to summarize that there are no genes particularly for SES and that the link between IQ and SES explains a quarter of the variance at best, and that the results are nested by a larger social context in which intelligence is economically valued.

These results could be exemplified with any young person entering adulthood. Based on Table 2, IQ heritability, that is cognitive ability-related genes (A) differing between individuals, played a substantive role, between 23% and 75%, in attaining education and occupation. The home environment (C), often thought to play a large role through resources and social connections, explained anywhere between 0% and 39%, often with deteriorating model fits. The rest of the variance (E) likely consisted of a myriad of tiny temporary experiences in life, such as being influenced by a friend or being at the right place at the right time, so called random events. This could for instance be the young person having aptitude for organizing his life, manifesting in high scores on IQ tests *and* entries into education and jobs.

The genetic impact aside, the results also point at environmental transmissions of cognitive ability on future SES, particularly on educational outcomes. A few of the univariate CTD models also indicated family environment variance (Table 2). This aligns with previous literature relating educational attainment to have a large share of common family variance, up to 40%, even though less so in extended twin models that include parents and siblings and control for assortative mating^7^. Interestingly, almost none of the explained variances in outcomes of occupation reported environmental linkages from IQ (Table 3).

### Study limitations

Some of the strengths of the present study can be attributed to the TwinLife project. The measurement (56 items in 4 domains) of IQ, and double measurements for both education and occupation, together with standardized procedures of collecting the data, empowered quality input data for the analyses. However, a non-trivial number of participants had no data particularly for the occupation prestige and occupation SES measures (Table S1), speculatively biasing the results. Yet, attrition analyses have concluded that dropout in the TwinLife data are mostly random^22^. Similarly, the predictive value and generalization of the study results should be questioned, seeing the limited time span of 4 years, from 23 to only 27 years of age. Arguably, there may not have been sufficient time nor enough waves of measurement for cognitive ability to fully shape future SES. Also, parental SES^10^ would have been a key control variable to further disentangle the effect of cognitive ability, however, somewhat outside the aim and scope of the present study.

Furthermore, most issues concerning genetically informed twin-studies such as the Equal Environment Assumption [23; 24], non-assortative mating, or minimum gene-environment interactions, will arguably always increase or decrease estimates, however, rarely overthrow results^25^. In the present sample, for instance, the estimate for heritability of cognitive ability was noticeably high (75%) for being a young adulthood sample. Embracing a skeptic attitude, it is worth remembering that the predictive variance from IQ on SES was generally phenotypically small, only 7—18% (see Table 1). Furthermore, reliability (internal instrument variance) in the IQ-composite is imperfect (α = 0.80), thus lowering the unique environmental variance (E) with SES according to the Cronbach’s alpha value.

Perhaps the principal weakness of the present study design is the oversimplification of decomposing variance into only genes (A) and environment (E); not exploring gene interactions (within-dominance or between-epistasis), twin-specific environment, passive gene-environment interactions, or gene-environment moderation effects. Genetically informed studies on the moderating effects of SES on IQ sometimes reveal so called hidden variance further complicating the narrative^24^. Still, the purpose of the present study results was to robustly and simplistically communicate psychological research not only to researchers, but also to the broad community and public.

## Conclusion and implications

Cognitive ability (IQ) has been widely researched before. However, it may not yet be as widely known that socioeconomic status outcomes are sometimes as heritable as individual IQ. The present study further adds to the existing knowledge by showing the directional (given the longitudinal models) linkage from IQ on SES, in the present study reporting multiple times more genetic than environmental explanations and overlaps. Such results may call for increased individual person-analysis both in research and policy interventions. Society tends to treat the population as one uniform mean value, particularly in political discourse and decision-making. The present results may also to a degree explain why societal distributions programs such as schooling facilities and support for the not so gifted or free higher education hasn’t reversed inequality in the job market noticeably. This may encourage new ways of thinking on how to counteract unwanted disparities in our future society; People *are* different – Genetic predispositions (i.e., individual differences) seem to play a role in individuals’ socioeconomic outcomes. Failure to account for these well-replicated genetic influences in research may present the wrong conclusions for both the public and academia.

## Supplementary Information

Below is the link to the electronic supplementary material.

Supplementary Material 1

## Data Availability

All TwinLife data is available on request, hosted at Leibniz Institute for the Social Sciences (GESIS.org). See [https://paneldata.org/twinlife/](https:/paneldata.org/twinlife) and soepmail@diw.de for data access.
