# Supplementary material for: Longitudinal associations between cognitive ability and socioeconomic status are partially genetic in nature
Source: Sci Rep. 2026 Feb 2;16:4315. doi: 10.1038/s41598-026-37786-3 (PMC12864940; doi:10.1038/s41598-026-37786-3)
Supplement: Supplementary file 1 — Supplementary Material 1 [file 41598_2026_37786_MOESM1_ESM.docx]

# Supplemental Materials

**Table S1.**

Descriptives Study Variables

|  | N | Mean | SD | Skewness | SE | Kurtosis | SE |
| --- | --- | --- | --- | --- | --- | --- | --- |
| IQ23_Cognitive ability | 787 | 39.28 | 6.94 | -0.69 | 0.09 | 0.31 | 0.17 |
| SES27_Edu. Level | 812 | 7.16 | 2.15 | -0.86 | 0.09 | -0.50 | 0.17 |
| SES27_Edu. Casmin | 812 | 7.02 | 1.91 | -0.73 | 0.09 | -0.12 | 0.17 |
| SES27_Occ. Prestige | 684 | 47.12 | 13.08 | 0.01 | 0.09 | -0.03 | 0.19 |
| SES27_Occ. SES | 583 | 3.35 | 2.60 | 1.03 | 0.10 | -0.44 | 0.20 |

**Table S2.**

Correlational Matrix Study Variables

|  |  | 1 | 2 | 3 | 4 | 5 |
| --- | --- | --- | --- | --- | --- | --- |
| 1.IQ23_Cognitive ability | Pearson's r | — |  |  |  |  |
|  | df | — |  |  |  |  |
|  | p-value | — |  |  |  |  |
|  | 95% CI Upper | — |  |  |  |  |
|  | 95% CI Lower | — |  |  |  |  |
| 2.SES27_Edu. Level | Pearson's r | 0.334*** | — |  |  |  |
|  | df | 709 | — |  |  |  |
|  | p-value | <.001 | — |  |  |  |
|  | 95% CI Upper | 0.398 | — |  |  |  |
|  | 95% CI Lower | 0.267 | — |  |  |  |
| 3.SES27_Edu. Casmin | Pearson's r | 0.435*** | 0.877*** | — |  |  |
|  | df | 709 | 730 | — |  |  |
|  | p-value | <.001 | <.001 | — |  |  |
|  | 95% CI Upper | 0.493 | 0.893 | — |  |  |
|  | 95% CI Lower | 0.374 | 0.860 | — |  |  |
| 4.SES27_Occ. Prestige | Pearson's r | 0.277*** | 0.426*** | 0.474*** | — |  |
|  | df | 602 | 621 | 621 | — |  |
|  | p-value | <.001 | <.001 | <.001 | — |  |
|  | 95% CI Upper | 0.349 | 0.489 | 0.533 | — |  |
|  | 95% CI Lower | 0.201 | 0.360 | 0.410 | — |  |
| 5.SES27_Occ. SES | Pearson's r | 0.312*** | 0.477*** | 0.482*** | 0.733*** | — |
|  | df | 513 | 530 | 530 | 530 | — |
|  | p-value | <.001 | <.001 | <.001 | <.001 | — |
|  | 95% CI Upper | 0.232 | 0.409 | 0.414 | 0.691 | — |
|  | 95% CI Lower | 0.388 | 0.540 | 0.545 | 0.770 | — |
